# Supplementary material for: Erythropoietin signaling regulates heme biosynthesis
Source: eLife. 2017 May 29;6:e24767. doi: 10.7554/eLife.24767 (PMC5478267; doi:10.7554/eLife.24767)
Supplement: Figure 1—source data 1. — Changes in the expression of proteins together with their accession numbers are shown in log2 scale. These data are depicted in the heat map in Figure 1C. DOI: http://dx.doi.org/10.7554/eLife.24767.004 [file elife-24767-fig1-data1.docx]

**Figure 1 – Source Data 1. Changes in the mitochondrial expression of select erythroid and housekeeping mitochondrial proteins.**

| **Protein** | **Accession** | **Fold Change (Log2)** |
| --- | --- | --- |
| PRKAR2B | NP_035288 | 2.226913852 |
| PRKACB | NP_001157670 | 2.006049899 |
| PRKACA | NP_001264827 | 1.931262586 |
| PRKAR1A | NP_068680 | 1.840898197 |
| PRKAR2A | NP_032950 | 1.023567883 |
| ABCB10 | NP_062425 | 2.396454481 |
| ATPIF1 | NP_031538 | 1.416763588 |
| FECH | NP_032024 | 1.221946223 |
| PPOX | NP_032937 | 1.145878137 |
| ALAS2 | NP_001095916 | 1.126147636 |
| CPOX | NP_031783 | 0.609230054 |
| MRPS18A | NP_081044 | -0.023904574 |
| MRPS11 | NP_080774 | -0.10199936 |
| MRPL32 | NP_083547 | 0.023511803 |
| MRPL18 | NP_080586 | -0.01040749 |
| TIMM17B | NP_035721 | 0.124735495 |
| TIMM22 | NP_062792 | 0.097606295 |
| TOMM22 | NP_766197 | 0.203265948 |
| TOMM6 | NP_079641 | 0.113481769 |
