# Supplementary material for: Erythropoietin signaling regulates heme biosynthesis
Source: eLife. 2017 May 29;6:e24767. doi: 10.7554/eLife.24767 (PMC5478267; doi:10.7554/eLife.24767)
Supplement: Figure 2—source data 1. — The increase in AKAP10 (NP_064305) mitochondrial expression is listed in log2 scale. These data are depicted in the heat map in Figure 2C. PRKAR2B and other controls were as previously shown in Figure 1C and Figure 1—source data 1. DOI: http://dx.doi.org/10.7554/eLife.24767.006 [file elife-24767-fig2-data1.docx]

**Figure 2 – Source Data 1. Change in the mitochondrial expression of AKAP10 during erythroid maturation.**

| **Protein** | **Accession** | **Fold Change (Log2)** |
| --- | --- | --- |
| AKAP10 | NP_064305 | 2.370640346 |
| PRKAR2B | NP_035288 | 2.226913852 |
| MRPS11 | NP_080774 | -0.10199936 |
| MRPL18 | NP_080586 | -0.01040749 |
| TIMM22 | NP_062792 | 0.097606295 |
| TOMM6 | NP_079641 | 0.113481769 |
