# Supplementary material for: Erythropoietin signaling regulates heme biosynthesis
Source: eLife. 2017 May 29;6:e24767. doi: 10.7554/eLife.24767 (PMC5478267; doi:10.7554/eLife.24767)
Supplement: Figure 5—source data 1. — The vmax and Km constants for unphosphorylated and in vitro phosphorylated FECH are shown. The data here are graphically presented in Figure 5B and Figure 5—figure supplement 1. *p-value<0.05, **non-significant, Mean ± SEM, n = 3. DOI: http://dx.doi.org/10.7554/eLife.24767.012 [file elife-24767-fig5-data1.docx]

**Figure 5 – Source Data 1. Maximum velocity (v_max_) and Michaelis-Menten (K_m_) constants.**

| **Protein** | **v_max_ (μM ^55^Fe/sec/μM FECH)*** | **K_m_ (μM ^55^Fe)**** |
| --- | --- | --- |
| FECH | 119.9 ± 4.4 | 1.731 ± 0.284 |
| pFECH | 153.3 ± 6.5 | 2.500 ± 0.493 |

**p*-value < 0.05, **non-significant
